# Clinical Effects of Balanced Crystalloids vs Saline in Adults With Diabetic Ketoacidosis: A Subgroup Analysis of Cluster Randomized Clinical Trials

**DOI:** 10.1001/jamanetworkopen.2020.24596

**Published:** 2020-11-16

**Authors:** Wesley H. Self, Christopher S. Evans, Cathy A. Jenkins, Ryan M. Brown, Jonathan D. Casey, Sean P. Collins, Taylor D. Coston, Matthew Felbinger, Lisa N. Flemmons, Susan M. Hellervik, Christopher J. Lindsell, Dandan Liu, Nicole S. McCoin, Kevin D. Niswender, Corey M. Slovis, Joanna L. Stollings, Li Wang, Todd W. Rice, Matthew W. Semler

**Affiliations:** 1 Department of Emergency Medicine, Vanderbilt University Medical Center, Nashville, Tennessee; 2 Department of Biostatistics, Vanderbilt University Medical Center, Nashville, Tennessee; 3 Asheville Pulmonary and Critical Care Associates, Asheville, North Carolina; 4 Division of Allergy, Pulmonary, and Critical Care Medicine, Vanderbilt University Medical Center, Nashville, Tennessee; 5 Department of Medicine, Vanderbilt University Medical Center, Nashville, Tennessee; 6 Department of Pharmacy Services, Vanderbilt University Medical Center, Nashville, Tennessee; 7 Division of Diabetes, Endocrinology, and Metabolism, Vanderbilt University Medical Center, Nashville, Tennessee; 8 Veterans Affairs Tennessee Valley Healthcare System, Nashville

## Abstract

**Importance:**

Saline (0.9% sodium chloride), the fluid most commonly used to treat diabetic ketoacidosis (DKA), can cause hyperchloremic metabolic acidosis. Balanced crystalloids, an alternative class of fluids for volume expansion, do not cause acidosis and, therefore, may lead to faster resolution of DKA than saline.

**Objective:**

To compare the clinical effects of balanced crystalloids with the clinical effects of saline for the acute treatment of adults with DKA.

**Design, Setting, and Participants:**

This study was a subgroup analysis of adults with DKA in 2 previously reported companion trials—Saline Against Lactated Ringer’s or Plasma-Lyte in the Emergency Department (SALT-ED) and the Isotonic Solutions and Major Adverse Renal Events Trial (SMART). These trials, conducted between January 2016 and March 2017 in an academic medical center in the US, were pragmatic, multiple-crossover, cluster, randomized clinical trials comparing balanced crystalloids vs saline in emergency department (ED) and intensive care unit (ICU) patients. This study included adults who presented to the ED with DKA, defined as a clinical diagnosis of DKA, plasma glucose greater than 250 mg/dL, plasma bicarbonate less than or equal to 18 mmol/L, and anion gap greater than 10 mmol/L. Data analysis was performed from January to April 2020.

**Interventions:**

Balanced crystalloids (clinician’s choice of Ringer lactate solution or Plasma-Lyte A solution) vs saline for fluid administration in the ED and ICU according to the same cluster-randomized multiple-crossover schedule.

**Main Outcomes and Measures:**

The primary outcome was time between ED presentation and DKA resolution, as defined by American Diabetes Association criteria. The secondary outcome was time between initiation and discontinuation of continuous insulin infusion.

**Results:**

Among 172 adults included in this secondary analysis of cluster trials, 94 were assigned to balanced crystalloids and 78 to saline. The median (interquartile range [IQR]) age was 29 (24-45) years, and 90 (52.3%) were women. The median (IQR) volume of isotonic fluid administered in the ED and ICU was 4478 (3000-6372) mL. Cumulative incidence analysis revealed shorter time to DKA resolution in the balanced crystalloids group (median time to resolution: 13.0 hours; IQR: 9.5-18.8 hours) than the saline group (median: 16.9 hours; IQR: 11.9-34.5 hours) (adjusted hazard ratio [aHR] = 1.68; 95% CI, 1.18-2.38; *P *= .004). Cumulative incidence analysis also revealed shorter time to insulin infusion discontinuation in the balanced crystalloids group (median: 9.8 hours; IQR: 5.1-17.0 hours) than the saline group (median: 13.4 hours; IQR: 11.0-17.9 hours) (aHR = 1.45; 95% CI, 1.03-2.03; *P *= .03).

**Conclusions and Relevance:**

In this secondary analysis of 2 cluster randomized clinical trials, compared with saline, treatment with balanced crystalloids resulted in more rapid resolution of DKA, suggesting that balanced crystalloids may be preferred over saline for acute management of adults with DKA.

**Trial Registration:**

ClinicalTrials.gov Identifiers: NCT02614040; NCT02444988

## Key Points

**Question:**

Does using a balanced crystalloid solution instead of saline for fluid therapy in adults with diabetic ketoacidosis (DKA) lead to faster resolution of DKA?

**Findings:**

In this subgroup analysis of 172 adults with DKA from 2 large cluster randomized clinical trials comparing balanced crystalloids and saline, the median time to DKA resolution was 13.0 hours with balanced crystalloids and 16.9 hours with saline, a significant difference.

**Meaning:**

These findings suggest that balanced crystalloid therapy leads to faster resolution of DKA than saline and may be the preferred isotonic fluid solution for acute management of DKA.

## Teaser

This subgroup analysis of 2 cluster randomized clinical trials examines adults presenting to the emergency department with diabetic ketoacidosis and determines how long it takes to reach resolution by treating with balanced crystalloids vs saline.

## Introduction

Along with insulin therapy, intravenous fluid administration to expand intravascular, interstitial, and intracellular volume is a key component of the acute management of diabetic ketoacidosis (DKA).^1,2,3^ Saline (0.9% sodium chloride, also called* normal saline*) is the most commonly used fluid for this purpose and the primary fluid recommended in current DKA clinical practice guidelines.^1,2,3^ The chloride concentration in saline (154 mmol/L) is higher than that in human plasma (94-111 mmol/L), which can cause hyperchloremic metabolic acidosis, especially when administered in large volumes.^4,5,6,7^ Although the clinical outcomes of saline infusion remain incompletely understood, accumulating evidence suggests that saline may increase the risk of kidney injury and impair recovery from severe illness, possibly because of the induction of metabolic acidosis.^8,9,10,11,12,13^

Balanced crystalloid solutions, including Ringer lactate and Plasma-Lyte A (Baxter Inc), contain chloride concentrations similar to those in human plasma and do not induce metabolic acidosis.^4,5,6,7^ Therefore, treatment of DKA with balanced crystalloids rather than saline may lead to faster resolution of DKA.^14,15,16,17^ However, balanced crystalloids also have theoretical risks in the treatment of DKA, including alkalosis and hyperkalemia, and the comparative effects of balanced crystalloids and saline in this setting are not well understood.^18^ In this subgroup analysis of 2 large pragmatic, cluster randomized clinical trials,^8,9^ we compared balanced crystalloids vs saline for the acute management of adults with DKA. The primary hypothesis was that balanced crystalloids would lead to more rapid resolution of DKA than saline.

## Methods

This study was a subgroup analysis of patients who presented to the emergency department (ED) with DKA within 2 recently completed companion pragmatic cluster trials—Saline Against Lactated Ringer’s or Plasma-Lyte in the Emergency Department (SALT-ED)^8^ and the Isotonic Solutions and Major Adverse Renal Events Trial (SMART).^9^ This secondary analysis was approved by the Vanderbilt University institutional review board with waiver of informed consent as an analysis of previously collected data. The relevant sections of the Consolidated Standards of Reporting Trials (CONSORT) reporting guideline were followed for this secondary analysis of a clinical trial.

### SALT-ED and SMART Trials

SALT-ED^8^ and SMART^9^ were cluster randomized clinical trials concurrently conducted at Vanderbilt University Medical Center to compare balanced crystalloids vs saline for intravenous fluid therapy in the ED and intensive care unit (ICU). Together, these trials enrolled 29 149 adult patients using broad eligibility criteria. In a cluster-randomized, multiple-crossover design, patients were assigned balanced crystalloids (clinician’s choice of Ringer lactate solution or Plasma-Lyte A) vs saline according to calendar month (eFigure 1 in the Supplement). Clinicians were instructed to use the assigned fluid type for fluid administration when volume expansion, fluid resuscitation, or an isotonic or near-isotonic fluid was desired. Assigned fluid type was controlled by the trial protocol in the ED, ICUs, and operating rooms, but not on the general hospital floors. Fluid assignment was the same in the ED, medical ICU, trauma ICU, and surgical ICU. Fluid assignment in 2 ICUs that admitted a low number of ED patients (neurologic ICU and cardiac ICU) was opposite of the ED (ie, these ICUs were assigned to saline when the ED was assigned to balanced crystalloids); these ICUs on an opposite schedule compared with the ED were not included in this DKA subgroup analysis. Data were collected from the electronic medical record. Data on race, ethnicity, and sex were based on patient self-report. Patient race was categorized as White or non-White. The non-White category included 46 Black participants and 2 participants who self-reported their race as neither Black nor White.

Patients treated in the ED and subsequently admitted to a general floor were analyzed in the SALT-ED trial.^8^ Patients treated in an ICU, including those initially treated in the ED and subsequently admitted to an ICU, were analyzed in the SMART trial.^9^ In both the SALT-ED and SMART trials, balanced crystalloids were associated with a lower incidence of major adverse kidney events than saline.^8,9^

### Population

The current study was a subgroup analysis of patients in the SALT-ED and SMART trials who presented to the study ED with DKA. All patients included in this study, regardless of whether they were originally analyzed in the SALT-ED population or the SMART population, presented to the ED with DKA and were part of the same multiple crossover allocation schedule for fluids. Inclusion criteria included (1) age 18 years or older; (2) presentation to the ED during the 15-month period when both the ED and ICUs were participating in the SALT-ED and SMART trials (January 1, 2016, to March 31, 2017); (3) a clinical diagnosis of DKA in the ED, defined as meeting both an *International Statistical Classification of Diseases, Tenth Revision, Clinical Modification (ICD-10-CM)* code for DKA (specific codes are listed in eTable 1 in the Supplement) and a medical record review confirming DKA was present at the time of ED evaluation rather than delayed onset of DKA in the hospital after admission; and (4) laboratory values in the ED consistent with DKA, including plasma glucose concentration greater than 250 mg/dL (SI conversion factor: to convert to millimoles per liter, multiply by 0.0555), plasma bicarbonate concentration less than or equal to 18 mEq/L (to convert to millimoles per liter, multiply by 1.0), and calculated anion gap (calculated as sodium concentration – [chloride concentration + bicarbonate concentration]) greater than 10 mEq/L (to convert to millimoles per liter, multiply by 1.0).^1^

Exclusion criteria included (1) transfer from an outside hospital to the study ED (these patients received intravenous fluid outside the study protocol prior to reaching the study ED); (2) admission to the cardiac or neurologic ICU (these units had the opposite crystalloid assignment schedule compared with the ED); and (3) presentation to the ED within 24 hours prior to a planned crossover in the trial (these patients experienced a crossover in the on-protocol crystalloid type during the first 24 hours of DKA treatment).

### Intervention

This subgroup analysis used the intervention delivered in the SALT-ED and SMART trials.^8,9^ Patients were assigned crystalloid type (balanced crystalloids vs saline) in the ED and ICU according to a cluster randomized, multiple crossover design in which the assigned crystalloid alternated each month (eFigure 1 in the Supplement). The intervention was delivered on a unit level by on-duty clinicians. Adherence to assigned crystalloid type was encouraged with automated electronic order entry algorithms, preferential stocking of the ED and ICUs with the on-protocol crystalloid type, and routine education, auditing, and feedback.^19,20^ Saline was the only on-protocol crystalloid for volume expansion during months assigned to saline. During months assigned to balanced crystalloids, clinicians had the option of selecting either Ringer lactate or Plasma-Lyte A, because both of these balanced solutions were available as part of routine care in the study hospital. Treating clinicians had the authority to override the trial protocol and deliver an off-protocol crystalloid if they believed it was indicated for a specific patient.

### DKA Management

The trial protocol only controlled the type of crystalloid use for volume expansion (balanced crystalloids vs saline). All other aspects of clinical management, including the volume of fluid administered, selection of other fluids (such as fluids with lower tonicity), and insulin dosing, were chosen by the treating clinicians without influence from the trial protocol. Recommended DKA management at the study hospital followed the American Diabetes Association Consensus Statement on the Management of Hyperglycemic Crises.^1^ This included administration of insulin by continuous intravenous infusion (insulin drip) to patients with an anion gap greater than 12 mEq/L, measurement of electrolytes every 2 hours during active DKA management, and discontinuation of the insulin infusion and transition to subcutaneous insulin when the anion gap decreased to less than or equal to 12 mEq/L. Blood samples for electrolyte measurements were collected by clinical personnel in lithium heparin vacutainers, and plasma concentrations were measured in the clinical laboratory. By hospital policy, patients receiving a continuous insulin infusion were managed in the ED or an ICU. Patients with DKA in the ED who did not require continuous insulin infusion after admission could be admitted to a general hospital floor outside the ICU according to the treating clinician’s discretion.

### Outcomes

The primary outcome was time to DKA resolution, defined as the time elapsed between ED presentation and resolution of ketoacidosis, using the following criteria for resolution of ketoacidosis from the American Diabetes Association Consensus Statement on Hyperglycemic Crises^1^—plasma glucose less than 200 mg/dL and 2 of the following: plasma bicarbonate greater than or equal to 15 mEq/L, venous pH greater than 7.3, and anion gap less than or equal to 12 mEq/L. Patients discharged from the hospital without meeting laboratory criteria for DKA resolution were classified as having DKA resolution at the time of hospital discharge.

The secondary outcome was time to discontinuation of insulin infusion, defined as the time between initiation and final discontinuation of intravenous insulin infusion during the hospitalization for DKA. Patients who never had an insulin infusion started were coded as 0 for time to discontinuation of insulin infusion. Additional outcomes are described in eTable 2 in the Supplement.

### Statistical Analysis

The primary analytical approach was an intention-to-treat analysis comparing outcomes between patients assigned to balanced crystalloids vs those assigned to saline. Plasma electrolyte concentrations during the first 72 hours of care after ED presentation were compared between groups using a univariate generalized additive model smoothing function.

Time to DKA resolution (primary outcome) included both right-censored data (death prior to DKA resolution) and left-censored data (DKA resolution defined by hospital discharge rather than laboratory criteria), and thus was analyzed using statistical methods for interval-censored outcomes. In an unadjusted analysis, a 2-sample weighted log-rank test was used to compare differences in cumulative incidence of DKA resolution between the balanced crystalloid group and saline group.^21^ Next, time to DKA resolution was evaluated using a multivariable Cox proportional hazards regression model in which the covariance matrices for the regression coefficients were estimated via 1000 bootstrap replications.^22,23^ The model included time to DKA resolution as the dependent variable, intervention group (balanced crystalloids vs saline) as the main independent variable, and the number of days elapsed between the beginning of the trial (January 1, 2016) and the patient’s ED presentation as a covariable. The trial included 1 cluster (all ED patients were on the same allocation schedule) and multiple periods. Hence, the model was not adjusted for clusters and was adjusted for period effects by including days elapsed since the beginning of the trial as a covariable.^8,19^ Adjusted hazards ratios (aHRs) were reported with the saline group serving as the referent, with an aHR greater than 1 indicating shorter time to DKA resolution for the balanced crystalloids group.

Time to discontinuation of insulin infusion (secondary outcome) included right-censored data (death prior to discontinuation). A Kaplan-Meier plot was used to illustrate the cumulative incidence of insulin infusion discontinuation with a log-rank test used to test the unadjusted difference between the balanced crystalloid group and saline group. A multivariable proportional hazards model was also constructed using the same approach as the primary outcome.

Additional outcomes were analyzed with multivariable regression models adjusting for time elapsed from trial initiation to ED presentation (eTable 2 in the Supplement).

Because this study is a subgroup analysis of completed clinical trials, the sample size was determined by the number of patients enrolled in the primary trials; power was not prospectively calculated for this analysis. The unit of analysis was each DKA hospitalization. While recognizing this study was a subgroup analysis and interpretation must consider this design, we considered a 2-sided *P* <.05 to denote statistical significance, and no adjustments were made for multiple testing. Analyses were completed with R statistical software version 3.5.2 (R Project for Statistical Computing) from January to April 2020.

## Results

### Population

During the 15-month study period, 271 patients treated in the ED received an *ICD-10-CM* code for DKA; 172 of these patients met the study eligibility criteria and were included in this analysis (eFigure 2 in the Supplement). Among the 172 patients in the study population, median (interquartile range [IQR]) age was 29 [24-45] years, 90 (52%) were women, 142 patients (83%) had type I diabetes, median (IQR) hemoglobin A_1c_ was 10.9% (9.2%-13.1%) (to convert to proportion of total hemoglobin, multiply by 0.01), median initial plasma bicarbonate concentration was 11 mEq/L, and a missed medication dose was the most common reason for DKA. Baseline characteristics in the balanced crystalloid group and saline group are presented in Table 1. The primary reasons for excluding patients who had an *ICD-10-CM* code for DKA were not meeting the laboratory case definition for DKA (65 patients) and transfer from an outside hospital (23 patients). According to the SALT-ED and SMART cluster-crossover schedule, 94 patients were assigned to balanced crystalloids during 8 months and 78 patients were assigned to saline during 7 months.

**Table 1.** Baseline Patient Characteristics by Assigned Treatment Group

Characteristic	Patients, No. (%)	
	Balanced crystalloids (n = 94)	Saline (n = 78)
Age, median (IQR), y	28 (23-39)	30 (25-49)
Women	54 (57.4)	36 (46.2)
Race		
White	66 (70.2)	58 (74.4)
Non-White	28 (29.8)	20 (25.6)
Elixhauser Comorbidity Index Score, median (IQR)^a^	5.0 (1.2-10.0)	5.0 (2.0-10.8)
Type of diabetes		
Type 1	82 (87.2)	60 (76.9)
Type 2	12 (12.8)	18 (23.1)
Baseline hemoglobin A_1c_, median (IQR), %^b^	10.8 (9.2-12.6)	11.3 (9.2-13.3)
Suspected cause of diabetic ketoacidosis		
Newly diagnosed diabetes	8 (8.5)	3 (3.8)
Missed medication dose(s)	52 (55.3)	42 (53.8)
Infection	16 (17.0)	24 (30.8)
Other^c^	10 (10.6)	6 (7.7)
Unknown	8 (8.5)	3 (3.8)
Diabetic ketoacidosis severity by initial plasma bicarbonate category in the ED		
Mild (15-18 mmol/L)	25 (26.6)	25 (32.1)
Moderate (10-14 mmol/L)	31 (33.0)	24 (30.8)
Severe (<10 mmol/L)	38 (40.4)	29 (37.2)
Initial Glasgow Coma Scale score in the ED <15	11 (11.7)	10 (12.8)
End-stage kidney disease with long-term kidney-replacement therapy at baseline	5 (5.3)	1 (1.3)
Baseline serum creatinine prior to acute illness, median (IQR), mg/dL^d^	0.70 (0.60-0.87)	0.74 (0.60-0.90)
Source of baseline creatinine prior to acute illness		
Measured value in medical record	70 (74.5)	52 (66.7)
Calculated value by equation	24 (25.5)	26 (33.3)
Stage 2 or greater acute kidney injury based on first creatinine measurement in ED^d^^,^^e^	56 (62.9)	44 (57.1)
Initial plasma laboratory values in the ED, median (IQR)		
Sodium, mEq/L	133 (129-135)	133 (130-136)
Chloride, mEq/L	98 (94-101)	97 (94-101)
Potassium, mEq/L	4.6 (4.1-5.1)	4.8 (4.4-5.5)
Bicarbonate, mEq/L	11 (7-15)	11 (7-16)
Creatinine, mg/dL^d^	1.5 (1.2-2.0)	1.5 (1.2-2.0)
Blood urea nitrogen, mg/dL^d^	18 (13-27)	20 (15-28)
Glucose, mg/dL	557 (415-761)	518 (373-750)
Anion gap, mEq/L	22 (19-28)	24 (19-29)

Abbreviations: ED, emergency department; IQR, interquartile range.

SI conversion factors: To convert anion gap to millimoles per liter, multiply by 1.0; bicarbonate to millimoles per liter, multiply by 1.0; chloride to millimoles per liter, multiply by 1.0; creatinine to micromoles per liter, multiply by 88.4; glucose to millimoles per liter, multiply by 0.0555; hemoglobin A_1c_ to proportion of total hemoglobin, multiply by 0.01; sodium to millimoles per liter, multiply by 1.0; potassium to millimoles per liter, multiply by 1.0; urea nitrogen to millimoles per liter, multiply by 0.357.

^a^ The Elixhauser Comorbidity Index score summarizes the burden of a patient’s coexisting conditions. Scores range from −19 to 89, with higher scores indicating a profile of coexisting conditions that is more strongly associated with in-hospital death.^24^

^b^ Eight patients had missing values for baseline hemoglobin A_1C_, including 5 patients in the balanced crystalloids group and 3 patients in the saline group.

^c^ Other suspected causes of DKA included pregnancy-related factors (3 patients in the balanced crystalloids group and 2 patients in the saline group), medication adverse effects (3 patients in the balanced crystalloids group), vomiting (2 patients in the balanced crystalloids group and 1 in the saline group), pancreatitis (1 patient in the balanced crystalloids group), gastrointestinal bleeding (1 patient in the balanced crystalloids group), heat exhaustion (1 patient in the saline group), trauma (1 patient in the saline group), and heart disease (1 patient in the saline group).

^d^ Six patients with baseline end-stage kidney disease with long-term kidney-replacement therapy were not included in the reported data for mean baseline serum creatinine prior to acute illness, source of baseline creatinine prior to acute illness, stage 2 or greater acute kidney injury in the ED, creatinine in the ED, and blood urea nitrogen in the ED.

^e^ Acute kidney injury was defined according to the Kidney Disease: Improving Global Outcomes creatinine criteria^25^: stage 2 or great acute kidney injury in ED defined as ED creatinine 200% or higher over baseline creatinine or ED creatinine greater than or equal to 4.0 mg/dL with an increase of at least 0.5 mg/dL over baseline creatinine.

### Fluid Administration

The selection of isotonic crystalloid type (balanced crystalloids vs saline) was controlled by the trial protocols in the ED and ICU. During treatment in the ED and ICU, the median (IQR) volume of isotonic crystalloids administered was 4478 (3000-6372) mL. Adherence to assigned crystalloid type was high (Table 2). By total volume, 85.3% of crystalloid administered in the balanced crystalloids group was balanced crystalloids and 96.7% of crystalloid administered in the saline group was saline. By volume, Ringer lactate solution accounted for 96.9% of balanced crystalloids used in this study, with Plasma-Lyte A accounting for the other 3.1%.

**Table 2.** Isotonic Crystalloids Received in the ED and ICU by Assigned Treatment Group

Variable	Balanced crystalloids (n = 94)	Saline (n = 78)
Total isotonic crystalloid volume, including both balanced crystalloids and saline, median (IQR), mL		
In the ED	2253 (1551-3000)	2106 (1901-3000)
In the ICU	2000 (0-4025)	2100 (0-3875)
Total in the ED and ICU combined	4267 (3000-7090)	4927 (3324-6026)
Balanced crystalloids volume, median (IQR), mL		
In the ED	2000 (1270-2755)	0 (0-0)
In the ICU	2000 (1000-4966)	0 (0-0)
Total in the ED and ICU combined	4000 (2312-6177)	0 (0-0)
Saline volume, median (IQR), m		
In the ED	0 (0-201)	2102 (1881-3000)
In the ICU	0 (0-0)	3000 (1800-4000)
Total in the ED and ICU combined	0 (0-788)	4694 (3324-5762)
Percentage of total isotonic crystalloid volume in the ED and ICU consistent with assigned group, patients, No. (%)		
100%	61 (65)	71 (91)
51%-99%	25 (27)	7 (9)
1%-50%	6 (6)	0
0%	2 (2)	0

Abbreviations: ED, emergency department; ICU, intensive care unit; IQR, interquartile range.

### Electrolyte Concentrations

**Figure 1.**
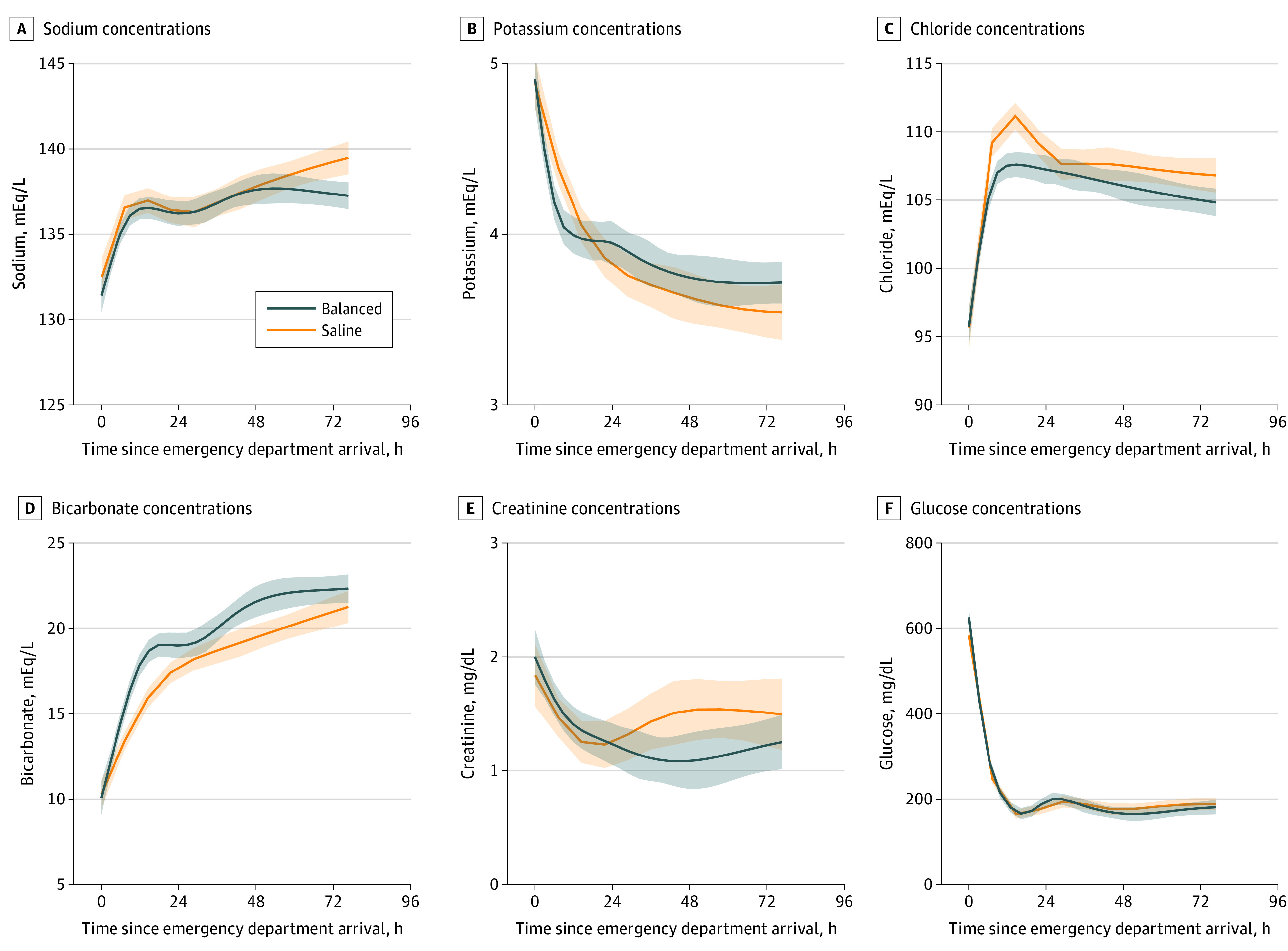
Plasma Electrolyte Concentrations in the First 72 Hours After Arrival in the Emergency Department by Assigned Treatment Group (Balanced Crystalloids vs Saline) Graphs show concentrations of sodium (A), potassium (B), chloride (C), bicarbonate (D), creatinine (E), and glucose (F). Lines denote means, and shaded bands denote 95% CIs. Plots were created with locally weighted scatterplot smoothing. Patients were censored at the time of hospital discharge or death. Separation of 95% CIs for chloride and bicarbonate suggest significant differences between the balanced crystalloids and saline groups. SI conversions: To convert bicarbonate to millimoles per liter, multiply by 1.0; chloride to millimoles per liter, multiply by 1.0; creatinine to micromoles per liter, multiply by 88.4; glucose to millimoles per liter, multiply by 0.0555; sodium to millimoles per liter, multiply by 1.0; potassium to millimoles per liter, multiply by 1.0.

Laboratory measurements needed to identify DKA resolution (glucose, bicarbonate, and anion gap, with or without pH) were completed at similar frequencies in the balanced crystalloids and saline groups. During the first 72 hours after ED presentation, the median (IQR) number of unique episodes in which all measurements to characterize DKA resolution were completed was 7 (5-10) in the balanced crystalloids group and 7 (5-10) in the saline group (*P* = .57). Plasma electrolyte concentrations during the first 72 hours after ED presentation are displayed in Figure 1. Compared with the saline group, patients in the balanced crystalloids group had lower chloride and higher bicarbonate concentrations over time after the initiation of treatment, as denoted by separation of 95% CIs between the 2 groups over time (Figure 1).

### Primary Analysis

**Figure 2.**
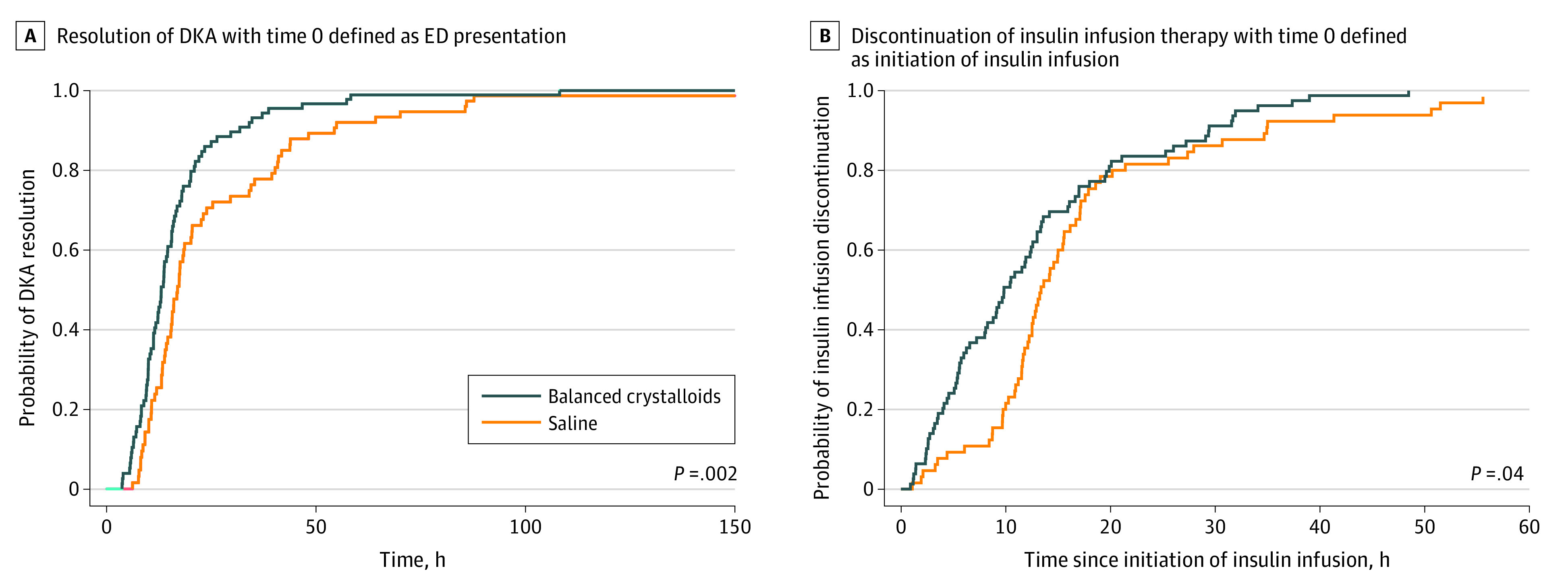
Cumulative Incidence by Assigned Treatment Group (Balanced Crystalloids vs Saline) of Resolution of Diabetic Ketoacidosis (DKA) and Discontinuation of Insulin Infusion Therapy Graphs show cumulative incidence of resolution of DKA (A) and discontinuation of insulin infusion therapy (B), with time 0 defined as emergency department (ED) presentation and initiation of insulin infusion, respectively. The cumulative incidence probabilities for DKA resolution were calculated using the Turnbull nonparametric method. Displayed *P* values were calculated with a log-rank test. Twenty-six patients (15 in the balanced crystalloids group and 11 in the saline group) were discharged from the hospital without meeting the laboratory definition of DKA resolution; these patients were considered to have DKA resolution at the time of hospital discharge.

Cumulative incidence analyses revealed that time to DKA resolution was shorter in the balanced crystalloids group (median [IQR] time to DKA resolution: 13.0 [9.5-18.8] hours) compared with the saline group (median [IQR]: 16.9 [11.9-34.5] hours) according to the unadjusted cumulative incidence (*P* = .002) (Figure 2A) and multivariable proportional hazards model (aHR = 1.68; 95% CI, 1.18-2.38; *P* = .004) (Table 3). Median (IQR) time to insulin drip discontinuation was shorter in the balanced crystalloids group (9.8 [5.1-17.0] hours) than in the saline group (13.4 [11.0-17.9] hours) according to the unadjusted cumulative incidence (*P* = .04) (Figure 2B) and multivariable proportional hazards model (aHR = 1.45; 95% CI, 1.03-2.03, *P* = .03) (Table 3). According to the median values, balanced crystalloids were associated with an absolute reduction of approximately 4 hours and a relative reduction of approximately 20% to 30% in the time to DKA resolution and discontinuation of insulin infusion.

**Table 3.** Outcomes by Assigned Treatment Group

Outcome^a^	Patients, No. (%)		Adjusted HR or OR (95% CI)^b^^,^^c^	Adjusted *P* value
	Balanced crystalloids (n = 94)	Saline (n = 78)		
Time to DKA resolution, median (IQR), h^d^	13.0 (9.5-18.8)	16.9 (11.9-34.5)	1.68 (1.18-2.38)	.004
Time to discontinuation of insulin infusion, median (IQR), h	9.8 (5.1-17.0)	13.4 (11.0-17.9)	1.45 (1.03-2.03)	.03
Continuous insulin infusion used	79 (84)	65 (83)	1.04 (0.46-2.35)	.92
ICU admission	77 (82)	65 (83)	0.65 (0.30-1.39)	.26
In-hospital death	0 (0)	1 (1)	Not calculated^e^	Not calculated^e^
Hospital-free days to day 28, median (IQR), d	26 (24-26)	26 (24 - 26)	1.13 (0.67-1.91)	.64
ICU-free days to day 28, median (IQR), d	27 (26-27)	27 (26 - 27)	1.16 (0.69-1.95)	.59
Stage 2 or greater acute kidney injury in hospital after ED	7 (8)	6 (8)	1.02 (0.33-3.17)	.98
Major adverse kidney events within 30 d	5 (5)	5 (6)	0.79 (0.22-2.86)	.72
New hyperkalemia (potassium >6.0 mmol/L) after ED presentation	11 (12)	18 (23)	0.51 (0.19-1.39)	.19
New hypokalemia (potassium <3.0 mmol/L) after ED presentation	9 (10)	15 (19)	0.35 (0.13-0.91)	.03
Seizure	1 (1)	2 (3)	0.40 (0.04-4.59)	.46
Lowest Glasgow Coma Scale score during hospitalization <15	29 (31)	22 (28)	1.24 (0.58-2.66)	.59
Invasive mechanical ventilation	3 (3)	2 (3)	1.24 (0.20-7.66)	.81

Abbreviations: DKA, diabetic ketoacidosis; ED, emergency department; HR, hazard ratio; ICU, intensive care unit; IQR, interquartile range; OR, odds ratio.

^a^ Definitions of each outcome are included in eTable 2 in the Supplement.

^b^ HRs were calculated for time to resolution of DKA, and time to discontinuation of continuous insulin infusion. ORs were calculated for the other outcomes.

^c^ All models were adjusted for days between the beginning of trial and ED presentation. In addition, the models for new hyperkalemia and hypokalemia were adjusted for initial ED plasma potassium concentration and the model for lowest Glasgow Coma Scale was adjusted for initial ED Glasgow Coma Scale score.

^d^ Twenty-six patients (15 in the balanced crystalloids group and 11 in the saline group) were discharged from the hospital without meeting the laboratory definition of DKA resolution and were considered to have DKA resolved at the time of hospital discharge.

^e^ Not calculated due to 0 outcomes in 1 of the groups.

Fewer patients experienced hypokalemia (potassium < 3.0 mmol/L) in the balanced crystalloids group than the saline group (adjusted odds ratio = 0.35; 95% CI, 0.13-0.91). Other clinical outcomes were similar between groups (Table 3). Most patients in both groups received continuous intravenous insulin infusion (79 patients [84%] in the balanced crystalloids group; 65 patients [83%] in the saline group). In both the balanced crystalloids group and saline group, the percentage of patients who experienced in-hospital death (0 patients vs 1 patients [1%], respectively), major adverse kidney events within 30 days (5 patients [5%] vs 5 patients [6%], respectively), and mechanical ventilation (3 patients [3%] vs 2 patients [3%], respectively) was low.

## Discussion

In this subgroup analysis of 2 cluster randomized clinical trials of adults presenting to the ED with DKA, treatment with balanced crystalloid solutions (largely lactated Ringer’s) was associated with more rapid resolution of DKA and discontinuation of insulin infusion than saline. According to the median values, balanced crystalloids were associated with an absolute reduction of about 4 hours and a relative reduction of approximately 20% to 30% in the time to DKA resolution and discontinuation of insulin infusion. There was no subgroup or outcome in which saline appeared superior to balanced crystalloids. These results suggest that balanced crystalloids may be preferred over saline for the acute management of adults with DKA.

A hallmark of DKA is the accumulation of ketone bodies (β-hydroxybutyrate and acetoacetate), resulting in an anion gap metabolic acidosis.^1^ Saline infusion leads to a nonanion gap hyperchloremic metabolic acidosis due to a supraphysiologic chloride concentration and strong ion difference of zero.^4,6,7^ As plasma chloride concentration increases, the other primary anion in plasma, bicarbonate, is lowered to maintain electroneutrality, which results in acidosis. Thus, treatment of DKA with saline could result in an iatrogenic acidosis that exacerbates metabolic acidosis caused by DKA itself. Balanced crystalloids contain physiologic concentrations of chloride and additional anions that metabolize into bicarbonate (sodium lactate in lactated Ringer’s and acetate in Plasma-Lyte A), which lead to neutral or mildly alkalizing effects when infused.^4,6,7,26^ These composition differences between balanced crystalloids and saline are the likely mechanism for shorter time to DKA resolution observed with balanced crystalloids in this study.

Current DKA clinical practice guidelines recommend saline as the fluid of choice for volume expansion,^1,2,3^ but they also acknowledge a paucity of evidence comparing saline with balanced crystalloids in this population.^2^ We believe that the results of this study add to the accumulating evidence suggesting balanced crystalloids are better resuscitation fluids than saline for many patients^8,9,11,12,27^ and may have particular benefits for patients with DKA.

The median reduction in time to DKA resolution with balanced crystalloids in our study was small. However, DKA is common.^1^ Saline and balanced crystalloids are both widely available and similar in cost,^28^ and the safety of administering fluids with a tonicity lower than saline in DKA is established.^29^ Therefore, no particular barrier exists to optimizing clinical care for DKA patients by incorporating use of balanced crystalloids. Consistent implementation of interventions that deliver small improvements in outcomes for common conditions can translate into substantial improvements in population health and health system function.

Four recent small trials compared balanced crystalloids and saline in patients with DKA.^15,16,17,30^ Consistent with our results, each of these trials found point estimates favoring faster DKA resolution with balanced crystalloids, although small sample sizes led to low power.^15,16,17,30^ Mahler et al^15^ randomized 45 adults with DKA and found Plasma-Lyte A led to higher plasma bicarbonate concentrations than saline after 24 hours (20 vs 17 mmol/L; *P* = .02). Van Zyl et al randomized 54 adults with DKA and found the median time to reach a pH greater than or equal to 7.32 was 540 minutes with lactated Ringer’s and 683 minutes with saline (*P* = .25). Yung et al^17^ randomized 77 children with DKA and found the geometric mean time to plasma bicarbonate concentration greater than or equal to 15 mmol/L was 6.2 hours with lactated Ringer and 8.6 hours with saline (*P* = .26). Williams et al^30^ randomized 66 children with DKA and found median time to resolution of DKA (defined as pH >7.3, bicarbonate >15 mmol/L, and normal sensorium) was 14.5 hours with Plasma-Lyte A and 16.0 hours with saline (*P* = .47). To our knowledge, our study adds to this literature as the largest study in the field.

### Strengths and Limitations

Our study had important strengths, including (1) immediate assignment of patients to balanced crystalloids vs saline upon ED presentation, which is often absent in trials with patient level randomization; (2) strong adherence with assigned fluid type; and (3) integration of the study intervention into ongoing clinical care conducted by on-duty clinicians, which should increase generalizability. This study had some limitations. First, this study was a subgroup analysis of prior clinical trials.^8,9^ Although we reported *P* values that have not been adjusted for multiplicity in this secondary analysis, a *P* value does not measure the importance or size of a result. We note that interpretation of findings should focus on effect sizes.^31^ Second, the intervention was not blinded. Third, this was a single-center study. Fourth, similar to prior trials,^16^ some patients did not meet the American Diabetes Association definition of DKA resolution^1^ before hospital discharge; we considered DKA to be resolved at the time of hospital discharge for these patients. Fifth, the study population was initially identified on the basis of *ICD-10-CM* codes, which could have resulted in some patients with DKA not being included in the study if DKA was not properly coded. Sixth, although crystalloid type was controlled by the trial in the ED and ICU, crystalloids administered on the hospital floors after transfer out of the ED and ICU was not controlled. Of note, by institutional policy, all patients were treated in the ED or ICU while receiving continuous insulin infusion; hence, most active DKA management was completed in the ED and ICU. Seventh, Ringer lactate solution was the predominant balanced crystalloid used in the study, and we have insufficient data to compare Ringer lactate with Plasma-Lyte A. Because Plasma-Lyte A has a greater alkalizing effect than Ringer lacate,^6,7^ we hypothesize that time to DKA resolution would be at least as fast with Plasma-Lyte as Ringer lactate. Eighth, although our sample size was larger than those in other trials in this field, the modest sample size led to some imbalances in baseline characteristics and limited our ability to robustly evaluate subgroups and rare clinical outcomes in DKA.

## Conclusions

In this subgroup analysis of adults with DKA within 2 large pragmatic trials, fluid administration with balanced crystalloids was associated with faster resolution of DKA than saline. These results suggest that balanced crystalloids may be preferred over saline for acute management of adults with DKA.

## Notes

Supplement. **eTable 1.** International Classification of Diseases, 10th Revision, Clinical Modification (ICD-10-CM) Codes for Diabetic Ketoacidosis (DKA) **eTable 2.** Study Outcomes **eFigure 1.** Sequence of Treatment Assignments for Balanced Crystalloids vs Saline in the SALT-ED and SMART Trials **eFigure 2.** Flow Diagram of Patient Participation
