# Supplementary material for: Clinical Effects of Balanced Crystalloids vs Saline in Adults With Diabetic Ketoacidosis: A Subgroup Analysis of Cluster Randomized Clinical Trials
Source: JAMA Netw Open. 2020 Nov 16;3(11):e2024596. doi: 10.1001/jamanetworkopen.2020.24596 (PMC7670314; doi:10.1001/jamanetworkopen.2020.24596)
Supplement: Supplement. — eTable 1. International Classification of Diseases, 10th Revision, Clinical Modification (ICD-10-CM) Codes for Diabetic Ketoacidosis (DKA) eTable 2. Study Outcomes eFigure 1. Sequence of Treatment Assignments for Balanced Crystalloids vs Saline in the SALT-ED and SMART Trials eFigure 2. Flow Diagram of Patient Participation [file jamanetwopen-e2024596-s001.pdf]

Supplemental Online Content

Self WH, Evans CS, Jenkins CA, et al. Pragmatic Critical Care Research Group. Clinical effects of balanced crystalloids vs saline in adults with diabetic ketoacidosis: a subgroup analysis of cluster randomized clinical trials. *JAMA Netw Open*. 2020;3(11):e2024596. doi:10.1001/jamanetworkopen.2020.24596

eTable 1. International Classification of Diseases, 10th Revision, Clinical Modification (ICD-10-CM) Codes for Diabetic Ketoacidosis (DKA)

eTable 2. Study Outcomes

eFigure 1. Sequence of Treatment Assignments for Balanced Crystalloids vs Saline in the SALT-ED and SMART Trials

eFigure 2. Flow Diagram of Patient Participation

This supplemental material has been provided by the authors to give readers additional information about their work.

Corresponding Author:
Wesley H. Self, MD, MPH; Department of Emergency Medicine; Vanderbilt University Medical Center; Email: wesley.self@vumc.org; Mail: 1313 21st Ave South; Oxford House 312; Nashville, TN 37232

© 2020 Self WH et al. *JAMA Network Open.*

eTable 1. International Classification of Diseases, 10th Revision, Clinical Modification (ICD-10-CM) codes for diabetic ketoacidosis (DKA). These codes were used as the initial step to identify patients with DKA treated in the study emergency department during the SALT-ED and SMART trials.

ICD-10-CM	Description
E08.10	Diabetes mellitus due to underlying condition with ketoacidosis without coma
E08.11	Diabetes mellitus due to underlying condition with ketoacidosis with coma
E09.10	Drug or chemical induced diabetes mellitus with ketoacidosis without coma
E09.11	Drug or chemical induced diabetes mellitus with ketoacidosis with coma
E10.10	Type 1 diabetes mellitus with ketoacidosis without coma
E10.11	Type 1 diabetes mellitus with ketoacidosis with coma
E11.10	Type 2 diabetes mellitus with ketoacidosis without coma
E11.11	Type 2 diabetes mellitus with ketoacidosis with coma
E13.10	Other diabetes mellitus with ketoacidosis without coma
E13.11	Other diabetes mellitus with ketoacidosis with coma

© 2020 Self WH et al. *JAMA Network Open.*

eTable 2. Study outcomes.

**Outcome**	**Definition and Analytical Approach**
Time to resolution of diabetic ketoacidosis (DKA)	Time between ED presentation and resolution of ketoacidosis, using the following criteria for resolution of ketoacidosis from the American Diabetes Association Consensus Statement on hyperglycemic crises[1]—plasma glucose <200 mg/dl and two of the following: plasma bicarbonate ≥15 mmol/l, venous pH >7.3, and anion gap ≤12 mmol/l. Anion gap was calculated as: sodium concentration – (chloride concentration + bicarbonate concentration)]. Sodium, chloride and bicarbonate concentrations were all measured from the same sample and reported in mmol/liter. Time to resolution of DKA was a continuous variable, measured in hours, and analyzed with a proportional hazards model.
Time to discontinuation of insulin infusion	Time between initiation and final discontinuation of intravenous insulin infusion ("insulin drip") during the index hospitalization for DKA; continuous variable; measured in hours; analyzed with a proportional hazards model.
Continuous insulin infusion used	Administration of insulin by continuous intravenous infusion at any time after ED presentation for the treatment of DKA; dichotomous; analyzed with a logistic regression model.
ICU admission	ICU admission at any time during the index hospitalization for DKA, including admission to an ICU directly from the ED, or initial general floor admission with later transfer to an ICU; dichotomous; analyzed with a logistic regression model.
In-hospital death	Death during the index hospitalization for DKA (death in the ED or in the hospital after admission); dichotomous; analyzed with logistic regression model.
Hospital-free days to day 28	Number of days alive and out of the hospital between ED presentation and 28 days later; continuous; measured in days, with a range from 0 to 28 days; analyzed with a proportional odds model. Calculated as: 28 days minus hospital length of stay. Patients who died during the index hospitalization were coded as having zero hospital-free days, which equated death to the worst possible length of stay. Patients discharged from the index hospitalization were assumed to survive outside the hospital through day 28; that is, rehospitalizations were not considered for the calculation of hospital-free days.
ICU-free days to day 28	Number of days alive and out of the ICU between ED presentation and 28 days later; continuous, measured in days, with a range from 0 to 28 days; analyzed with a proportional odds model. Calculated as: 28 days minus ICU length of stay. Patients who died during the index hospitalization were coded as having zero ICU free days, which equated death to the worst possible ICU length of stay. If a patient had multiple ICU stays within the index visit for DKA, all ICU stays were included in the calculation of ICU free-days. Patients discharged from the index hospitalization were assumed to survive outside the ICU through day 28; that is, rehospitalizations were not considered for the calculation of ICU-free days.
Stage 2 or greater acute kidney injury in hospital after ED	Fulfillment of at least one of the following three criteria based on creatine values measured after the initial value in the ED and before the earlier of hospital discharge or day 30: maximum plasma creatinine concentration at least 200% of the baseline value; an increase in the plasma creatinine concentration to ≥4 mg/dL with an absolute increase of ≥0.5 mg/dL; or initiation of new renal-replacement therapy. Dichotomous variable; analyzed with a logistic regression model. This definition is

© 2020 Self WH et al. *JAMA Network Open.*

	consistent with creatinine criteria for Stage 2 or greater acute kidney from the Kidney Disease: Improving Global Outcomes (KDIGO) group.[28] Baseline creatinine was defined as the lowest recorded value within the electronic medical record at the study institution in the year prior to ED presentation. Patients with no recorded creatinine values in the prior year had a baseline creatinine value calculated assuming normal baseline renal function using the following equation: [creatinine = 0.74 – 0.2 (if female) + 0.08 (if Black) + 0.003 x age (in years)]. Patients with end stage renal disease on chronic renal replacement therapy at the time of ED presentation were not eligible for the acute kidney injury outcome. Acute kidney injury based on initial creatine values measured in the ED is considered a baseline characteristic (present before the intervention). Acute kidney injury based on creatinine values measured in the hospital after the initial ED values is considered an outcome (ascertained after the intervention).
Major Adverse Kidney Events within 30 days (MAKE30)	Composite outcome of: (i) death, (ii) new renal replacement therapy, or (iii) final serum creatinine ≥ 200% of baseline at the earliest of hospital discharge or 30 days after ED presentation; dichotomous; analyzed with a logistic regression model. Patients meeting any of the three components were coded as meeting the MAKE30 outcome. Baseline creatinine was defined as the lowest recorded value within the electronic medical record at the study institution in the year prior to ED presentation. Patients with no recorded creatinine values in the prior year had a baseline creatinine value calculated assuming normal baseline renal function using the following equation: [creatinine = 0.74 – 0.2 (if female) + 0.08 (if Black) + 0.003 x age (in years)]. Patients with end stage renal disease on chronic renal replacement therapy at the time of ED presentation were eligible for the MAKE30 outcome through the death component only; these patients were not eligible for new renal replacement therapy or persistent renal dysfunction.
New hyperkalemia (K >6.0 mmol/liter) after ED presentation	Plasma potassium concentration >6.0 mmol/liter at any time during the index hospitalization for DKA after the initial ED value; dichotomous; analyzed with a logistic regression model adjusted for initial potassium concentration in the ED. Patients with an initial ED plasma potassium concentration >6.0 mmol/liter were not eligible for this outcome.
New hypokalemia (K <3.0 mmol/liter) after ED presentation	Plasma potassium concentration <3.0 mmol/liter at any time during the index hospitalization for DKA after the initial ED value; dichotomous; analyzed with a logistic regression model adjusted for initial potassium concentration in the ED. Patients with an initial ED plasma potassium concentration <3.0 mmol/liter were not eligible for this outcome.
Seizure	Seizure identified by the clinical team and documented in the electronic medical record at any time during the index hospitalization for DKA; dichotomous; analyzed with a logistic regression model.
Lowest Glasgow Coma Scale during hospitalization <15	Recorded Glasgow Coma Scale (GCS) value <15 during the index hospitalization after the initial ED; dichotomous; analyzed with a logistic regression model adjusted for initial GCS score in the ED
Invasive mechanical ventilation	Initiation of new invasive positive pressure mechanical ventilation through an endotracheal tube or tracheostomy at any time during the index hospitalization for DKA; dichotomous; analyzed with a logistic regression model. Patients who presented to the ED already receiving invasive mechanical ventilation were not eligible for the invasive mechanical ventilation outcome.

© 2020 Self WH et al. *JAMA Network Open.*

Changes in plasma electrolyte concentrations during initial 72 hours of care after ED presentation	Plasma electrolyte measurements obtained by clinical teams during the first 72 hours following ED arrival were plotted. Comparisons between the balanced crystalloid group and saline group were conducted using a univariate generalized additive model smoothing function.

© 2020 Self WH et al. *JAMA Network Open.*

eFigure 1. Sequence of treatment assignments for balanced crystalloids versus saline in the SALT-ED[8] and SMART trials[9]. Treatment assignment alternated each calendar month between balanced crystalloids (BC) and saline (S) in each unit participating in the trial. The current analysis of patients with diabetic ketoacidosis (DKA) was limited to months when both the emergency department (ED) and medical intensive care unit (MICU) were active in the trials – January 2016 through March 2017.

	2015							2016												2017			
	Jun	Jul	Aug	Sep	Oct	Nov	Dec	Jan	Feb	Mar	Apr	May	Jun	Jul	Aug	Sep	Oct	Nov	Dec	Jan	Feb	Mar	Apr
**ED**								BC	S	BC	S	BC	S	BC	S	BC	S	BC	S	BC	S	BC	S
**Medical ICU**	S	BC	S	BC	S	BC	S	BC	S	BC	S	BC	S	BC	S	BC	S	BC	S	BC	S	BC	
**Neuro ICU**					BC	S	BC	S	BC	S	BC	S	BC	S	BC	S	BC	S	BC	S	BC	S	
**Cardiac ICU**							BC	S	BC	S	BC	S	BC	S	BC	S	BC	S	BC	S	BC	S	
**Trauma ICU**										BC	S	BC	S	BC	S	BC	S	BC	S	BC	S	BC	S
**Surgical ICU**												BC	S	BC	S	BC	S	BC	S	BC	S	BC	S

DKA Analysis: January 1, 2016 – March 31, 2017

© 2020 Self WH et al. *JAMA Network Open.*

eFigure 2. Flow diagram of patient participation.

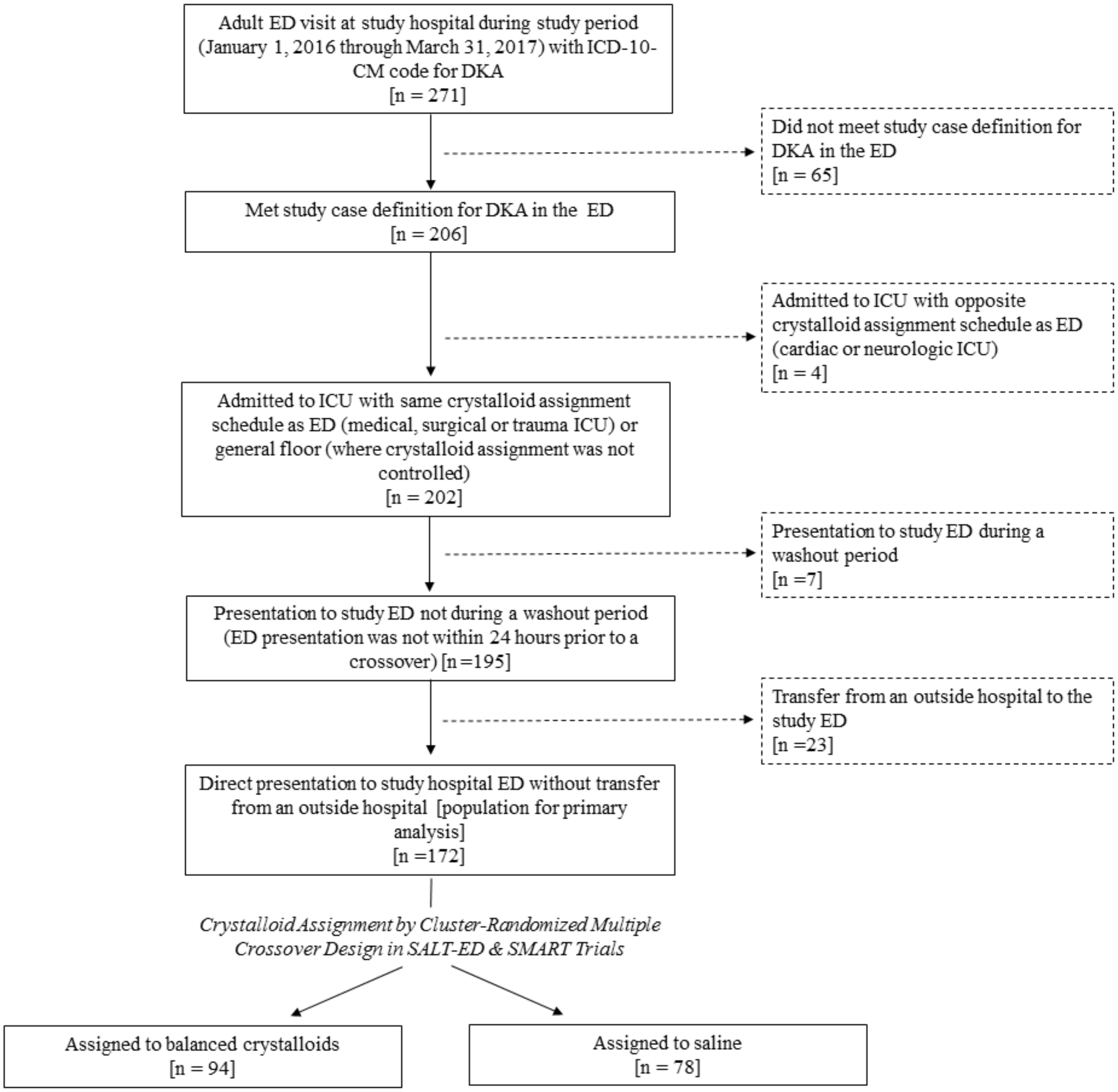

© 2020 Self WH et al. *JAMA Network Open.*
